# Folic acid and cancer risk in BRCA1 carriers

**DOI:** 10.1186/1897-4287-10-S3-A6

**Published:** 2012-04-20

**Authors:** Katarzyna Durda, Anna Jakubowska, Krzysztof Kąklewski, Katarzyna Jaworska, Satish Gupta, Tomasz Byrski, Tomasz Huzarski, Jacek Gronwald, Cezary Cybulski, Tadeusz Dębniak, Aleksandra Tołoczko, Oleg Ashuryk, Jan Lubiński

**Affiliations:** 1 Pomeranian Medical University, Clinic of Oncology, Szczecin, Poland; 2 Postgraduate School of Molecular Medicine, Warsaw Medical University, Warsaw, Poland

## 

Recent studies indicate that the selected micro-and macro-elements and vitamins may significantly influence the risk of cancer. Folic acid is necessary for DNA synthesis and repair, plays an important role in one-carbon groups for methylation reactions. The MTHFR gene produces an enzyme important in folate metabolism.

The aim of study was to analyze an association of folic acid concentrations and genetic variants in the MTHFR gene with breast cancer risk in patients with BRCA1 mutation.

Study group consisted of 99 patients with breast or ovarian cancer and 198 healthy women from the paired control group; all cases and controls were carriers of one out of three Polish founder mutations (C61G, 4153delA, 5382insC). Folic acid concentration was quantitatively measured in blood plasma by HPLC chromatography (Flexar HPLC, Perkin Elmer). Two functional SNPs in the MTHFR gene, 677 C>T (rs1801133) and 1298 A>C (rs1801131), both associated with reduced enzyme activity, have been tested on LightCycler 480 (Roche Diagnostic) by Taqman or Simple Probe approach.

The mean levels of folic acid were compared for cases and controls. Women who have level of folic acid in the range of 53.3 mg/l - 83.1 mg/l had a significantly lower risk of breast or ovarian cancer (OR = 0.55, p = 0.0087) – comparison to quartile with the lowest concentration. The analysis of correlation between the level of folic acid and tested genetic variants in the MTHFR gene gave the following results:

- for carriers of MTHFR 1298AA the lowest risk of cancer was for folic acid concentration >81 mg/l (OR = 0,25, p = 0,0292 , CI = 0,07948-0,7863)

**Table 1:** 

FOLATE [mg/]	CASES	CONTROLS	OR	p-value
3,34 - 34,28	13	17	0,77	0,79
34,5 - 53,44	15	15	1,0	-
53,75 - 80,92	8	22	0,36	0,11
81,07 - 284,79	6	24	0,25	0,03

**-** for carriers of MTHFR 1298AA +AC the lowest risk of cancer was for folic acid concentration in the range of 51,44 to 82-82 mg/l (OR = 0,25, p = 0,009, CI = 0,09-0,67)

**Table 2:** 

FOLATE [mg/]	CASES	CONTROLS	OR	p-value
4,92-30,83	20	20	1,0	-
31,36-51,29	13	26	0,50	0,17
51,44-82,82	8	32	0,25	0,01
82,94-195,81	15	24	0,62	0,37

